# Communications

**Published:** 1873-05

**Authors:** 


*Communications.*

The following communications have been received: "Clinical Report of Stray Cases of Constitutional Syphilis," by T. Curtis Smith, M.D., of Ohio; "Medical Observations," by Fred Horner, Jr., M.D., of Virginia. We thank *all* our friends for their valuable articles. We will publish as promptly as possible. Send on your articles. Don't stop writing. We and the profession wish to hear from you.

